# Supplementary material for: Thrombospondin-4 controls matrix assembly during development and repair of myotendinous junctions
Source: eLife. 2014 Jun 18;3:e02372. doi: 10.7554/eLife.02372 (PMC4096842; doi:10.7554/eLife.02372)
Supplement: Supplementary file 1. — List of primer sequences used for qRT-PCR, cloning of mRNA and probe synthesis. DOI: http://dx.doi.org/10.7554/eLife.02372.023 [file elife02372s001.docx]

**Supplementary File 1. List of primer sequences used for qRT-PCR, cloning of mRNA and probe synthesis**

| **Name** | **Sequence** | **Gene** |
| --- | --- | --- |
| lama2-fp-qpcr | ccaacccagaaaagggaaac | *laminin a2* |
| lama2-rp-qpcr | tgaactccagatccactgacac |  |
| lamb2-fp-qpcr | tggcacagatccagtcagag | *laminin b2* |
| lamb2-rp-qpcr | tgttgttggcacgtttgg |  |
| lamc1-fp-qpcr | caatcaatgcactgctaggc | *laminin c1* |
| lamc1-fp-qpcr | ttgagagcgttgtcgatctg |  |
| lamc2-fp-qpcr | atcgctgacaacatgcagac | *laminin c2* |
| lamc2-fp-qpcr | tggtcatgccctctagtttg |  |
| tsp4b-ntd-5’-XhoI | **ctgagctcgag**acgtcaccggactgtctacc | *tsp4b* N-terminal region for epitope cloning. Bold region encodes restriction enzyme linker to facilitate cloning. |
| tsp4b-ntd-3’-NotI | **ctgagctcgag**gacctttgttagctccagca |  |
| tsp4b-probe-fp | cttaacgtcaccggactgtctac | *tsp4b* probe for in situ hybridization. Bold region encodes T7 promoter. |
| tsp4b-probe-rp-t7 | **taatacgactcactataggg**gcaaatgtacttgcattctaggc |  |
| tsp4b-sig-fp-SalI | **cagtcgacta**atggccggcacaatgcatctcc | *tsp4b* full length cDNA Bold region encodes restriction enzyme linker to facilitate cloning. |
| tsp4b-XbaI-rp | **tagttctaga**ttacaaggggtccatgccatg |  |
| Tnmd-FP | atcattctgagtgtggtgttcct | *tnmd* probe for in situ hybridization. Bold region encodes T7 promoter. |
